# Proteomics and Its Current Application in Biomedical Area: Concise Review

**DOI:** 10.1155/2024/4454744

**Published:** 2024-02-17

**Authors:** Semira Gobena, Bemrew Admassu, Mebrie Zemene Kinde, Abebe Tesfaye Gessese

**Affiliations:** ^1^College of Veterinary Medicine and Animal Sciences, University of Gondar, Gondar, Ethiopia; ^2^Department of Veterinary Biomedical Sciences, College of Veterinary Medicine and Animal Sciences, University of Gondar, Gondar, Ethiopia

## Abstract

Biomedical researchers tirelessly seek cutting-edge technologies to advance disease diagnosis, drug discovery, and therapeutic interventions, all aimed at enhancing human and animal well-being. Within this realm, proteomics stands out as a pivotal technology, focusing on extensive studies of protein composition, structure, function, and interactions. Proteomics, with its subdivisions of expression, structural, and functional proteomics, plays a crucial role in unraveling the complexities of biological systems. Various sophisticated techniques are employed in proteomics, including polyacrylamide gel electrophoresis, mass spectrometry analysis, NMR spectroscopy, protein microarray, X-ray crystallography, and Edman sequencing. These methods collectively contribute to the comprehensive understanding of proteins and their roles in health and disease. In the biomedical field, proteomics finds widespread application in cancer research and diagnosis, stem cell studies, and the diagnosis and research of both infectious and noninfectious diseases. In addition, it plays a pivotal role in drug discovery and the emerging frontier of personalized medicine. The versatility of proteomics allows researchers to delve into the intricacies of molecular mechanisms, paving the way for innovative therapeutic approaches. As infectious and noninfectious diseases continue to emerge and the field of biomedical research expands, the significance of proteomics becomes increasingly evident. Keeping abreast of the latest developments in proteomics applications becomes paramount for the development of therapeutics, translational research, and study of diverse diseases. This review aims to provide a comprehensive overview of proteomics, offering a concise outline of its current applications in the biomedical domain. By doing so, it seeks to contribute to the understanding and advancement of proteomics, emphasizing its pivotal role in shaping the future of biomedical research and therapeutic interventions.

## 1. Introduction

The dynamic role of biomolecules in human and animal life has been documented since the inception of biological research [1, 2]. However, possessing knowledge of the structure and nucleotide sequence of genes is not sufficient to comprehensively illustrate the overall activities within living organisms. Therefore, there is a growing emphasis on understanding gene products, known as proteins, using proteomics-based technology [3]. Proteomics involves the study of all proteins expressed in a cell or organism, with a focus on their composition, structure, function, interaction, expression profiling, and modifications [4].

Proteomics offers a superior understanding of an organism's structure and function compared to genomics, despite its greater complexity, as protein expression undergoes changes over time and in response to environmental conditions [5]. Relying solely on the study of genes makes it impossible to acquire various types of information. For example, elucidating the mechanisms behind disease development, aging, and the impacts of environmental factors is not achievable solely through genome studies. Moreover, the identification of drug targets and the characterization of protein modifications are possible only through the examination of proteins [6]. Consequently, it is increasingly crucial to comprehend how proteins within a cell interact with each other and how these interactions respond to both internal and external signals [3].

Proteomics-based technologies find application in diverse biomedical contexts, including the detection of diagnostic markers, understanding pathogenesis, observing changes in protein expression patterns in response to internal or external signals, and interpreting functional protein pathways in various diseases [2]. They also play a crucial role in drug discovery and the identification of candidate vaccines by pinpointing proteins that can serve as drug and vaccine targets [7].

Currently, a variety of proteomics techniques are employed, including gel electrophoresis, chromatography, microarray, mass spectrometry, and Edman sequencing [2].

As infectious and noninfectious diseases continue to emerge and biomedical research expands, the use of proteomics is steadily increasing. Consequently, keeping abreast of the latest developments in proteomics is likely to significantly impact drug and vaccine development, translational research, and serve as the foundation for the study of various diseases in the future [8]. Therefore, the primary objective of this review is to provide an overview of proteomics and offer a concise outline of current techniques and applications in the biomedical field.

## 2. Proteomics

Proteomics involves the examination of the proteome, which refers to the complete collection of expressed proteins within a cell. More specifically, proteomics encompasses the processes of identifying and quantifying proteins, as well as determining their localization, composition, structures, functions, interactions, expression profiling, and modifications [4]. This field holds significant importance in biomedical research, particularly in deciphering disease pathogenesis and prognosis, diagnosing diseases, and providing the foundation for the discovery of biologics [3, 8–10].

### 2.1. Types of Proteomics

Proteomics can be categorized into expression proteomics, structural proteomics, and functional proteomics, depending on how proteins respond under stress conditions [11, 12].

#### 2.1.1. Expression Proteomics

Expression proteomics involves the qualitative and quantitative examination of the overall protein expression differences between samples influenced by a specific factor [11]. This factor might encompass a disease, a drug treatment, or an environmental condition. For instance, this approach facilitates the comparison of protein expression across the entire proteome or subproteomes in normal and diseased cell samples. In addition, this method can unveil novel proteins involved in signal transduction or pinpoint disease-specific proteins [13]. Techniques such as 2D polyacrylamide gel electrophoresis, mass spectrometry, and microarrays are employed to identify disparities in protein expression among samples [11, 12].

#### 2.1.2. Structural Proteomics

The study of proteomics, aimed at elucidating the 3D structure and intricate structural features of functional proteins found within a specific cellular organelle, is referred to as “cell mapping” or structural proteomics [11]. In this discipline, the identification and localization of entire proteins within a complex system or organelles are undertaken, and potential protein-protein interactions are determined [14]. Furthermore, structural proteomics enables the comprehension of drug target proteins through structural analysis. For instance, it is employed in the structural analysis of the nuclear pore complex [15]. The primary methods for conducting structural proteomics are X-ray crystallography and nuclear magnetic resonance (NMR) spectroscopy [11].

#### 2.1.3. Functional Proteomics

Functional proteomics investigates protein functions, molecular mechanisms within a cell, and interactions among protein partners. The identification of an unknown protein associating with partners from a specific protein complex involved in a particular mechanism strongly indicates its biological function [16].

## 3. Techniques Used in Proteomics

### 3.1. 1D and 2D Polyacrylamide Gel Electrophoresis

One-dimensional (1D) polyacrylamide gel electrophoresis is a technique used to separate protein mixtures on a polyacrylamide gel based on their molecular mass after being solubilized in sodium dodecyl sulfate (SDS). The separated proteins are then extracted from the gel for further characterization, such as determining amino acid sequences and posttranslational protein modifications. However, due to the limited resolving power of a 1D gel, especially when dealing with more complex protein mixtures like crude cell lysates, two-dimensional (2D) gel electrophoresis can be employed [17].

Two-dimensional polyacrylamide gel electrophoresis, first described by O'Farrell in 1975, has evolved significantly as a core technology for analyzing complex protein mixtures extracted from biological samples. 2D PAGE is a high-resolution technique that separates proteins based on charge and mass. The gel is run in one direction in a pH gradient under nondenaturing conditions to separate proteins by isoelectric points (pI). Then, in an orthogonal dimension under denaturing conditions, proteins are separated by molecular weights (MW). The outcome is a two-dimensional gel map with small spots, each corresponding to a single expressed protein. 2D gel electrophoresis is primarily used to compare two similar samples to identify specific proteins [18]. It possesses an unparalleled ability to separate thousands of proteins simultaneously and is unique in its capacity to resolve post and cotranslational modifications that cannot be predicted from the genome sequence [4, 19].

Despite its utility in resolving complex protein mixtures, 2D gel electrophoresis has limitations in terms of reproducibility, the detection of low-abundance and hydrophobic proteins, and low sensitivity in identifying proteins with pH values that are too low (pH < 3) or too high (pH < 10), as well as molecular masses that are too small or too high [20]. In addition, gel electrophoresis possesses challenges in analyzing membrane proteins, which are predominantly hydrophobic and not easily solubilized [4].

### 3.2. Mass Spectrometry Analysis

Mass spectrometry (MS) serves as a high-throughput analytical detection method that can determine the molecular weights and chemical structures of peptides, proteins, carbohydrates, oligonucleotides, natural products, and drug metabolites [21]. The operation of the spectrometer relies on the separation of molecules based on their mass-to-charge (m/z) ratio, achieved through high-energy electron ionization that breaks molecules into smaller fragments [22, 23]. The entire process involves three steps: first, converting biomolecules in a liquid or solid phase into gas-phase ions; second, separating these ions using m/z values in a mass analyzer compartment under the influence of magnetic or electric fields; and finally, measuring the separated ions and quantifying each species with a specific m/z value [24].

Two widely used ionization techniques are electrospray ionization (ESI) and matrix-assisted laser desorption ionization (MALDI) [24]. Both processes involve adding or removing protons to transform peptides into ions. The “soft” ionization techniques, such as ESI and MALDI, allow ion production without significant compromise to sample integrity, enabling precise mass data collection for proteins and peptides in their natural forms [6]. While MALDI results in both positive and negative ionization for different molecule types, ESI generates multiple charged ionized molecules, particularly beneficial for high molecular mass and thermally unstable compounds such as proteins, oligonucleotides, and synthetic polymers [25–27].

MALDI stands out due to its automation potential, allowing a robot to apply samples, and its widespread sensitivity compared to other laser ionization methods [25, 26]. Samples can often be used directly after in-gel digestion without the need for purification, providing an advantage over ESI [28]. On the other hand, ESI offers strong repeatability and flexibility to handle various MS categories, with the ability to be applied to quadrupole, ion traps, time-of-flight (TOF)-MS, and Fourier transform ion cyclotron resonance. However, ESI has limitations, including its inability for molecular imaging, high sample requirements, and the complexity of MS/MS spectra, due to the production of numerous peaks from various charged ions [29].

According to Zhu and Fang [30] and Glish and Vachet [31], MS offers several advantages, such as low sample requirements, label-free detection, quick analysis, the ability to define chemical structures through fragmentation, high sensitivity, and simultaneous detection of multiple analytes. Due to these benefits, MS is commonly employed for various molecular biology analysis goals, either independently or in conjunction with other structural proteomics techniques [32, 33]. Examples of analyses performed include molecular weight characterization, identification of posttranslational modifications in proteins, vibrational component identification in proteins, analysis of conformation and dynamics of proteins, detection of noncovalent interactions, protein and peptide sequencing, DNA sequencing, protein folding, in vitro drug analysis, and drug discovery [31, 34].

#### 3.2.1. Matrix-Assisted Laser Desorption/Ionization-Time-of-Flight Mass Spectrometry

The MALDI device serves as the ionization source, and the TOF functions as the mass analyzer when used in tandem in the MALDI-TOF mass spectrometer. Various application areas include identifying cancer biomarkers in various cancers [35], characterizing microorganisms such as bacteria, fungi, and viruses [36], and analyzing glycoproteins, oligonucleotides, carbohydrates, and small biomolecules [37].

#### 3.2.2. Liquid Chromatography-Mass Spectrometry

The method called liquid chromatography-mass spectrometry, or LC-MS, combines sample separation through LC and analysis via MS. LC technology, with its capability to handle large and delicate biomolecules, allows the analysis of proteins extracted from complex mixtures. When coupled with MS, LC-MS can identify peptides within the mixture [38]. Due to its ability to modify proteins, LC aids researchers in discovering new biomarkers and understanding the mechanisms underlying cancer development. For example, some researchers use LC-MS/MS to swiftly monitor congenital adrenal hyperplasia using dried filter-paper blood samples [39].

In various application areas, such as biopharmaceutical drug development, drug metabolism, toxicology studies, drug quantification in biological fluids, pharmacokinetic studies, bioavailability studies, doping control, quantification of biogenic amines, and therapeutic drug monitoring, LC-MS serves as a bioanalytical method for the quantitative analysis of proteins. The linkage of liquid chromatography to tandem mass spectrometry (LC-MS/MS) proves to be a successful bioanalytical method for determining protein-based biopharmaceuticals in biological matrices [40, 41].

#### 3.2.3. Tandem Mass Spectrometry

Tandem mass spectrometry, commonly referred to as MS/MS, is a two-step method for analyzing a sample. This involves either a single mass spectrometer with multiple analyzers placed sequentially or the connection of two or more mass spectrometers. TANDEM MS (MS/MS) is composed of a TOF analyzer with two or three quadrupoles [41]. The specific type of MS/MS analysis achievable depends on the mass analyzer connected to the MALDI source [42].

MS/MS proves particularly valuable when evaluating complex mixtures, as it integrates two phases of MS. In the initial step of MS/MS, a predetermined set of m/z ions is separated from other ions originating from the ion source and fragmented through a chemical process. Mass spectra are then generated for these fragments in the second stage. TANDEM MS is commonly employed in drug bioanalysis, where it is used in conjunction with HPLC to identify and quantify both phase I and phase II drug metabolites [31].

#### 3.2.4. Gas Chromatography-Mass Spectrometry (GC-MS)

In 1950s, gas chromatography was developed by Roland Gohlke and Fred McLafferty, who utilized a mass spectrometer as the detector. This technique combines the benefits of mass spectrometry with gas-liquid chromatography to identify various compounds in a test sample. GC-MS finds applications in diverse fields such as identifying unknown materials, environmental analysis, drug detection, explosives investigation, and fire investigation. In addition, it can be utilized in airport security to detect drugs on individuals or in luggage. Moreover, materials once thought to have decomposed beyond recognition can have trace elements identified through GC-MS. Due to its application in conducting specific tests, GC-MS is often acclaimed as the “gold standard” for forensic substance identification. A positive result from a particular test confirms the presence of a specific material in the sample. GC-MS is also employed to determine metabolic activity in conjunction with isotope labeling of metabolic products. The majority of applications involve labeling and ratio measurements utilizing an isotope ratio mass spectrometer [43].

### 3.3. NMR Spectroscopy

Nuclear magnetic resonance (NMR) stands out as an excellent method for examining the molecular structure, folding, and function of proteins. The process of identifying structures through NMR spectroscopy typically involves multiple steps, each requiring a set of highly specialized methods. To validate the structure, samples undergo processing, measurements are taken, and interpretive techniques are then applied. According to Wiese et al. [44], protein structure holds crucial significance in various research fields, including homology modeling, structure-based drug design, and functional genomics.

An example of NMR application is the establishment of the three-dimensional structure of the transmembrane domain of outer membrane protein A from *Escherichia coli*, utilizing heteronuclear nuclear magnetic resonance in dodecylphosphocholine micelles. This 19 kDa (177 amino acids) protein fold comprises of an eight-stranded *β*-barrel connected by tight twists on the periplasmic side and larger mobile loops near the extracellular side [45]. Another study using NMR examined the interaction between iso-1-cytochrome c and yeast cytochrome c peroxidase, revealing chemical shifts for both 1H and 15N nuclei [46].

Holmes et al. utilized NMR spectroscopy to explore differences in metabolic phenotypes across 4,630 individuals from 4 human populations. The examined metabolic phenotypes resulted from the interplay of various factors, including nutritional, environmental, genetic, and gut microbial activities. Significant links were found between blood pressure, urine metabolites, and selective metabolites in various populations, offering the potential for the discovery of new biomarkers [47].

NMR can be effectively combined with other techniques such as LC or UHPLC to enhance the sensitivity and resolution of high-throughput protein profiling. In addition, the generation of structural information is evaluated concerning the identification of metabolites in complex mixtures [48]. In the context of identifying potential biomarkers for early diagnosis and prognosis, the combination of ultra-high performance liquid chromatography (UHPLC) with NMR was developed to elucidate metabolic abnormalities in patients with esophageal cancer. This study revealed significant differences in amino acid and lipid metabolism, as well as in ketogenesis, glycolysis, and the tricarboxylic acid cycle when comparing esophageal cancer patients to controls [49].

### 3.4. X-Ray Crystallography

X-ray crystallography stands as the preferred method for elucidating the three-dimensional structure of proteins. Following exposure to X-rays, highly pure crystallized samples undergo diffraction, and the resulting diffraction patterns allow the determination of the size of the repeating crystal unit and crystal packing symmetry. The applications of X-ray crystallography are diverse, encompassing the study of immunological complexes, protein-nucleic acid complexes, and viral systems. Furthermore, the three-dimensional protein structure provides comprehensive insights into drug design, site-directed mutagenesis, protein-ligand interactions, and the clarification of enzyme mechanisms [50].

The critical components of the spatial ring structure promoting *E. coli* cell division are ZipA and FtsZ. The interaction between FtsZ, a homolog of eukaryotic tubulin, and ZipA, a membrane-anchored protein, is mediated by C-terminal domains. Through X-ray crystallography, the structure of the FtsZ C-terminal segment and the FtsZ-ZipA binding complex was determined [51]. Similarly, X-ray crystallography was employed to unveil the structure of the Norwalk virus, causing human gastroenteritis. The results revealed a viral capsid consisting of 180 repeating units of a single protein, connected by a flexible hinge. The shell (S) domain displayed an eight-stranded *β*-sandwich pattern, while the protruding (P) domain showed structural similarities to the eukaryotic translation elongation factor domain, influencing strain specificity and cell binding according to Prasad et al. [52].

Lipid transfer proteins facilitating the transfer of phospholipids, glycolipids, steroids, and fatty acids (nsLTPs) are not specific to a particular membrane. The comparative structure of maize nsLTP in complex with various ligands revealed variations in the volume of the hydrophobic cavity based on the size of bound ligands [53]. In humans, the drug-drug interactions that induce or inhibit enzymes metabolically clearing clinically utilized medicines are catalyzed by microsomal cytochrome P450 3A4. Examination of the protein structure using X-ray crystallography uncovered a substantial substrate binding cavity capable of oxidizing large substrates such as taxanes, cyclosporin, statins, and macrolide antibiotics [54]. The three-dimensional structure of recombinant horseradish peroxidase in combination with benzohydroxamic acid (BHA) was also revealed through X-ray crystallography, showcasing BHA's electron density in the peroxidase active site and the adjacent hydrophobic pocket [55].

### 3.5. Protein Microarray

Protein chips, also known as protein microarrays, represent a novel category of proteomics methods capable of rapidly generating substantial data with minimal sample amounts. As outlined by Sutandy et al. [56], protein microarrays can be categorized into three types: analytical protein microarrays, functional protein microarrays, and reverse-phase protein microarrays.

#### 3.5.1. Analytical Protein Microarray

The prevalent form of analytical protein microarray is the antibody microarray, where proteins are recognized through direct protein labeling subsequent to antibody capture. These arrays are commonly employed for measuring protein expression levels and binding affinities [56–58]. A high-throughput proteome analysis of cancer cells was conducted using an antibody microarray to identify differentially expressed proteins in tissues obtained from oral cavity squamous carcinoma cells [59]. In addition, protein profiling of bladder cancer was accomplished using antibody arrays [60]. Microarray immunoassays were employed to detect *Bacillus globigii*, ricin, cholera toxin, and staphylococcal enterotoxin B [61]. For the identification of cellular signaling networks and characterization of plant kinases using protein microarrays, both analytical and experimental methods have been established [62]. Arabidopsis mitogen-activated protein kinases (MAPKs) have been elucidated, highlighting their ubiquity and high conservation in plants, where they respond to a wide range of extracellular stimuli [63].

#### 3.5.2. Functional Protein Microarray

Functional protein microarrays are generated using purified proteins, facilitating the exploration of diverse interactions, including those involving proteins and their substrates, drugs, proteins, and DNA [56]. Functional protein microarrays have played a crucial role in describing the functions of thousands of proteins. For instance, research on the protein-protein interaction in *A. thaliana* led to the discovery of calmodulin-like proteins (CML) and substrates of calmodulin (CaM) [64].

#### 3.5.3. Reverse-Phase Protein Microarray

Cell lysates from various cell phases are arranged on nitrocellulose slides and probed with antibodies specific to target proteins. The antibodies are then detected using colorimetric, chemiluminescent, and fluorescent assays. Slides are equipped with reference peptides for protein quantification. According to Sutandy et al. [56], these microarrays are employed to identify dysfunctional or altered proteins indicative of a particular disease. For the large-scale analysis of phosphorylation states and protein expression in human stem cells and acute myelogenous leukemia cells, reverse-phase protein microarray analysis of hematopoietic stem cell and primary leukemia samples has proven highly reproducible and reliable [65]. The reverse-phase protein microarray technique was evaluated in non-small cell lung cancer (NSCLC) cell lines for the quantitative analysis of phosphoproteins and other cancer-related proteins, tracking apoptosis, DNA damage, cell-cycle control, and signaling pathways [66].

### 3.6. Edman Sequencing

Edman sequencing is a technique used to determine the sequence of amino acids in peptides and proteins. This method utilizes chemicals to react and extract the amino acid residues present at the N-terminus of the polypeptide chain, playing a crucial role in assessing the quality of therapeutic proteins and biopharmaceuticals [6].

### 3.7. Bioinformatics in Proteomics

Cutting-edge proteomics algorithms are employed in bioinformatics analyses to handle the vast and diverse data involved in the process of marker discovery [67]. Despite the challenges associated with managing these extensive data and identifying connections across various omics technologies such as genomics and metabolomics, bioinformatics strives to navigate through. Difficulties arise from factors involved in processing, quality assessment, and the lack of data format standards in proteomics data analysis. The main challenge lies in analyzing large datasets to derive genuine biological insights [9].

Protein pathways, which are internal cellular processes, each exert distinct biological effects. Several resources and databases offer information on protein pathways, including the Kyoto Gene and Genome Encyclopedia, BioCarta, and Pathway Knowledge Base. Comprehensive data on metabolism, signaling, and interactions can be found in databases like Reactome and Ingenuity pathway [68, 69]. Recent signal transduction pathway databases such as GenMAPP and Protein Analysis Through Evolutionary Relationships (PANTHER) have been developed [70]. Specialized databases, such as NetPath, which contains pathways linked to cancer, have been established to identify proteins specific to particular cancer types [71].

For information about protein interactions in complexes, databases such as BioGRID, IntAct, MINT, and HRPD provide valuable resources [72–74]. The STRING database is widely utilized for studying protein interactions and is interconnected with various other databases for literature mining. In addition, the STRING database enables the creation of protein networks based on the provided gene list and available interactions [75, 76].

## 4. Application of Proteomics

Proteomics is presently applied in various contexts, and some of these are outlined. Protein profiles, levels, sites of modification, and interactions in pathological conditions are just a few of the domains where proteomics yields valuable information [8].

### 4.1. Cancer Research and Diagnosis

Proteomics has emerged as a valuable scientific technique for investigating molecular changes in cancer. This approach has been instrumental in the identification of therapeutic targets [8, 77] and biomarkers with potential clinical applications. It offers crucial insights into the molecular aspects of tumor growth and metastasis [8].

The term “oncoproteomics” refers to the application of proteomics in cancer research [78]. This has enabled the discovery of protein expression patterns and biomarkers that contribute to tumor categorization, prognosis, and prediction, as well as the identification of potential therapy responders. In clinical practice, the glycoprotein antigen, for instance, is frequently assessed as a tumor marker for epithelial ovarian tumors. It is used to monitor prognosis, track disease progression, and enhance care for women diagnosed with ovarian cancer. Microarray technology and laser capture microdissection (LCM) of tumor tissue are employed to categorize proteins in cancer [79].

Oncoproteomics finds applications in various tissues such as the brain, colon, breast, rectum, and prostate. Proteomics not only aids in discovering new treatments but also in identifying different types of cancer [80]. Among the proteomics approaches available for finding cancer biomarkers are aptamer-based molecular probes, cancer immunomics, tissue microarrays, nano-proteomics (for identifying autoantibody signatures), and antibody microarrays [78]. It is noteworthy that databases containing the cancer proteome have recently been established and are openly accessible through bioinformatics integration [8].

### 4.2. Stem Cell Study

Proteomics provides the most effective approach to address the numerous unanswered questions in both basic and clinically oriented stem cell research. For instance, the identification of differentiation-specific proteins, which could serve as biomarkers for intermediate or terminal stages of cell differentiation or aid in distinguishing tumorigenic cells from the overall cell population, remains largely unknown. Similarly, the cell-surface proteins and signaling cascades of stem cells and their differentiated progenies are areas where understanding is lacking [81]. The discovery of proteins like colony-stimulating factors (CSFs) and cell-surface CD molecules has resulted in significant advancements in hematopoietic stem cell (HSC) research. Mass spectrometry-based proteomics has been instrumental in studying various developmental processes, including spermatogenesis [82], lineage specification [83], and brain differentiation [84].

### 4.3. Autoimmune Disease Diagnosis

Profiling of autoantibody responses can be undertaken by utilizing biological fluids obtained from patients afflicted with autoimmune diseases, and proteomics technologies are proven to be highly beneficial in this endeavor. Proteomics techniques play a crucial role in discerning auto-reactive B-cell responses in conditions such as rheumatoid arthritis, multiple sclerosis, and autoimmune diabetes. These techniques further enable the categorization of individual patients based on their unique “autoantibody fingerprint.” The profiling of autoantibodies and the phenomenon of epitope spreading have contributed to the identification and characterization of autoantigens, thereby enhancing the potential for antigen-specific therapy [85].

### 4.4. Cardiovascular Diseases

The global incidence of stroke and heart failure is on the rise, and these severe conditions are associated with unfavorable outcomes and a grim prognosis [86]. Several studies have utilized various heart failure models and proteomic approaches, including LC-MS/MS linked iTRAQ [87] and MALDI-MS/MS combined with DIGE [88], to explore the molecular pathways involved. As a result of these investigations, a well-recognized pathway model has been established, encompassing mitochondrial electron transport machinery, TCA components, glycolysis, and fatty acid metabolism [89]. However, proteomic indicators for heart failure with direct therapeutic applications remain elusive.

In a study employing SELDI-TOF-MS, Scott et al. [90] identified six potential serum biomarkers, though none proved predictive of a patient's suitability for therapeutic intervention. Kuznetsova et al. [91] examined urine biomarkers in hypertension using CE-MS and, after correcting for multiple testing, revealed a peak pattern consisting of 85 discriminatory peaks. Three potential biomarkers were distinct enough to differentiate individuals with essential hypertension and left ventricular diastolic dysfunction from healthy controls. Despite these promising initial findings, the therapeutic relevance remains uncertain as the molecular identity of the suggested biomarkers and their validation through other methods have yet to be established.

### 4.5. Kidney Diseases

Glomerulonephritis, a leading cause of renal failure and a significant contributor to morbidity and mortality in kidney diseases with multiple etiologies, is a primary concern [92]. The diagnosis of glomerular disease involves assessing proteinuria, evaluating renal function, and analyzing urine through microscopy and dipstick tests. While these findings are useful for identifying kidney damage, they may not effectively detect underlying or concurrent inflammatory diseases. Due to this limitation, various potential biomarkers associated with glomerular disease, such as growth factors, chemokines, and cytokines, have been investigated. Recently, mass spectrometry (MS) has been employed to screen individuals at different stages of the disease, as well as animal models, for urine biomarkers in glomerulonephritis [93].

Similarly, in immunoglobulin A nephropathy, several urine biomarkers identified using capillary electrophoresis-mass spectrometry (CE-MS) and 2D-gel techniques were suggested to differentiate glomerulonephritis from other proteinuric glomerular disorders [94]. Notably, Haubitz et al. [95] observed changes in peptide peak patterns excretion with the increasing use of antihypertensive medications, implying that urinary biomarkers might be employed in the future to monitor the effectiveness of clinical drug treatment for glomerulonephritis. This is supported by an independent 2005 study by Rossing et al., which found that treating patients with diabetic nephropathy with candesartan, an angiotensin II receptor blocker, significantly reduced the excretion of disease-specific biomarkers, approaching levels observed in urine from healthy controls [96].

Diabetic nephropathy is a major contributor to morbidity and mortality in patients with both type 1 and type 2 diabetes mellitus. While microalbuminuria is a critical early indicator of this condition, a significant glomerular impairment often occurs by the time it becomes apparent. Rossing et al. [96] used MS to identify less pronounced patterns of microalbuminuria in diabetes and urine polypeptide patterns in normoalbuminuria patients. This suggests the possibility of constructing a predictive model to identify patients at risk of renal injury before the onset of nephropathy.

### 4.6. Neurological Complexes

Undoubtedly, the human brain and its peripheral circuits constitute one of the most intricate and poorly comprehended biological systems in humans. A significant breakthrough occurred in 2000 when Husi et al. discovered a vast and dense molecular network of synaptic proteins, incorporating at least 250 proteins into a substructure known as the postsynaptic density [97]. While many of these proteins were recognized as crucial components of learning and memory, the groundbreaking aspect was the demonstration, unprecedented in neuroscience or any other system, of the direct association and preassembly of pathways and signaling machinery to such an extent [98]. This led to the hypothesis that complex illnesses like Parkinson's and Alzheimer's might result from network disturbances, where unrelated protein or gene damage produced similar effects [99].

Protein biomarker discovery from serum and other biological fluids, utilizing MS-based methodologies and related technologies for identifying protein biomarker signatures, has been applied to neurodevelopmental diseases, including attention deficit hyperactivity disorder [100], autism spectrum disorder [101], Alzheimer's disease [102], Parkinson's disease [103], neuropsychiatric disorders like schizophrenia [104], and neurodegenerative conditions like multiple sclerosis [105]. Despite having limited information in proteome pattern analysis, the latter two conditions are associated with changes in metabolic patterns, including serotonin levels. Nevertheless, research has shown a high correlation between latrophilin LPHN3 expression levels and attention deficit hyperactivity disorder at the proteome level [106].

Specifically, various MS-based techniques have been employed to identify potential biomarkers for multiple sclerosis. Stoop et al. [107] utilized peptide and protein profiling to discern differences between multiple sclerosis patients and controls. Subsequently, MALDI-MS was employed for a semiquantitative analysis of differentially abundant proteins in a follow-up investigation [108]. In a 2010 study, Comabella et al. utilized iTRAQ analysis on cerebrospinal fluid from individuals with multiple sclerosis, revealing differences in the expression of 23 proteins between those with worsening multiple sclerosis and those with stable conditions. One of the differentially expressed proteins, Chitinase-3-like 1, was independently verified using ELISA [109]. Another investigation by Mattsson et al. in 2007, using cerebrospinal fluid from multiple sclerosis patients, reported a significantly altered pattern of 24 proteins, including the downregulation of chromogranin B and secretogranin II [110].

### 4.7. Infectious Disease Diagnosis

Proteomics is increasingly vital for the identification of pathogens, detection of emerging and reemerging infectious agents, understanding pathophysiology, and diagnosing diseases. The integration of metagenomics, proteomics, and metabolomics has significantly contributed to the exploration and comprehension of bacterial physiology [111]. Recently, proteomics technologies have revolutionized vaccine development and disease diagnosis. More precise and time-efficient technologies now facilitate the prompt identification of infections, leading to early diagnoses. Mass spectrometry, by combining various gel-based or shotgun proteomics techniques, has enhanced the depth and thoroughness of information on the proteome of any toxic substance. MALDI-ToF has proven highly beneficial in the identification and differentiation of bacterial pathogens. Other quantitative methodologies are being explored for investigating bacterial virulence factors, diagnostic markers, and vaccine candidates. Proteomics offers the advantage of identifying secreted proteins and understanding their roles in virulence [112]. Moreover, proteomics techniques are employed to study oxidative stress, the function of proteins in host-pathogen interactions, the concealed processes of infections, and the identification of the proteins involved [113].

### 4.8. Drug Discovery

The process of discovering new drugs is complex and involves various steps, including functional, chemical, and clinical proteomics-based methods. Proteomics has broadened its application in drug development to encompass patient therapy and care [114]. Due to its incapacity to separate membrane proteins, which constitute around 50% of significant therapeutic targets, 2-DE is not a useful method for drug discovery [115]. In addition, 2-DE cannot detect low-abundance proteins [114]. Understanding the role of individual proteins and their interactions within a mixture is crucial for proteomics of drug discovery. This understanding facilitates the identification of pharmacological targets, the development of more potent medications, and the evaluation of the effects of those medications on patients [116]. The techniques must be capable of identifying low-abundance proteins and their activity. Phage proteins have been identified and separated using various technologies, including MS and protein-chips. Other methods for the same objective include activity-based assays and two-hybrid assays [117]. *Lavandula angustifolia* was used as a medication to treat Alzheimer's disease in rats, employing 2D-PAGE MALDI-TOF/TOF [118].

Recently, advances in proteomics and computational approaches have significantly reduced the time and resource requirements for chemical production and biological testing [119]. This enables researchers to screen a large number of proteins in clinically distinct samples, aiding in the identification of disease biomarkers, the identification or validation of drug targets, the design of more potent medications, and the evaluation of drug efficacy and patient response, among other steps in the current drug discovery process [116]. The detection of cell death in cells and tissues holds immense therapeutic potential [120].

Proteomics analysis is performed to explore the correlation between the structure and function of fibroin proteins found in silk fibers, which show significant promise as biomaterials. Silk-based biomaterials are primarily recognized for their suitability in biomedical and tissue engineering applications, including drug delivery and the creation of implantable devices, due to their biocompatibility and favorable physical and chemical properties. Leveraging the knowledge of silk's biological properties in wound healing or treating abrasions is anticipated to enhance healing applications given the current circumstances [121, 122]. For instance, the regenerative capacity of the adult brain is significantly limited following injuries such as trauma and stroke. Researchers have addressed this issue by developing injectable hydrogel scaffolds based on 3D silk fibroin, encapsulating neural stem cells to promote brain regeneration. To enhance the hydrogel's functionality for neural stem cells, silk fibroin was modified through the conjugation of an IKVAV peptide. The impact of this modification on cell viability and neural differentiation was evaluated, revealing that IKVAV-modified silk fibroin hydrogels exhibited improved cell viability and enhanced neural differentiation capability. These modified hydrogels, incorporating IKVAV, are utilized in brain tissue engineering [123].

Moreover, proteomics is employed to characterize the properties of peptide and protein-based hydrogel biomaterials, extensively applied in biomedical fields such as tissue engineering, wound therapy, and drug delivery [123, 124].

### 4.9. Personalized Medicine

The advancement of advanced modern technology for thorough monitoring and evaluation of patient DNA, RNA, protein, and metabolite data has driven the evolution of personalized medical care. Therapeutic decision-making now relies on a few validated and approved molecular diagnostics [124, 125]. However, for personalized molecular medicine to fully realize its potential, several aspects need improvement, including robustness, cost-effectiveness, wide applicability, and regulatory compliance. A major challenge lies in effectively managing and processing the vast volumes of data generated using high-throughput techniques, aiming to enhance data visualization, downstream analysis, and the integration of multi-OMICS approach data. Moreover, there is a scarcity of computational tools for this type of multidimensional data analysis, knowledge expansion, and construction, despite their essential role in achieving better patient outcomes.

Systems biology, as an iterative approach, aims to describe and visualize interrelated events within cells, tissues, and entire systems. It integrates foundational knowledge from biology and medicine with insights from other scientific disciplines. By explaining how various molecular shifts lead to modulated events and, ultimately, altered cell behavior under disease conditions, systems biology addresses impacts such as environmental and network connectivity (e.g., gene pattern shifts, modulated signaling cascades, runaway or inhibited metabolic pathways). A cross-disciplinary effort of this nature is required to comprehend and transform multidimensional datasets into actionable information that can categorize and stratify disease states. This effort is crucial for identifying potential unique biomarkers usable in prognosis, diagnosis, and therapeutic agent response. To prevent unintended side effects and adverse events and avoid unsuccessful therapies on a personalized basis, a comprehensive understanding of regulated events using targeted medicines on various biological and pharmacological levels is necessary. Systems biology, by identifying critical components in aberrant pathways, molecular drivers, and biomarkers, has the potential to introduce novel regimes in clinical trial design. This approach can target subpopulations of disease cases where the benefits of a targeted approach are not yet evident, potentially accelerating the adoption of successful treatments in the clinical setting and increasing the overall success rate [126].

## 5. Challenges and Limitations in Proteomics

Studying proteins presents numerous distinct challenges, with the primary obstacle being the significant variation in protein expression depending on the cell type and environment [127]. In addition, unlike genomics, there is no equivalent polymerase chain reaction (PCR) approach for proteomics [128]. For example, analyzing low-abundance proteins remains a significant challenge as there is no PCR equivalent for proteins, and these proteins may not always be detected due to the typical loss of 30–40% of proteins during sample preparation [129]. Moreover, preserving the native conformations of proteins is essential for obtaining meaningful results in protein interaction research.

The highly regulated posttranslational nature of protein activity further complicates proteomics [130]. The types of samples and methods used for sample preparation can significantly impact the quality of mass spectrometry (MS) data. For instance, the choice of biospecimen and sample processing method affected the levels of protein and phosphoprotein in breast cancer tumor samples [131].

Despite the rapid development of protein analysis technologies, examining proteins on the same scale as nucleic acids remains a challenge. The majority of proteomics relies on non-high-throughput techniques like PAGE or protein purification. Even with MS, data collection or analysis can be time-consuming. While a MALDI-TOF mass spectrometer can swiftly and automatically analyze hundreds of proteins, the compromised data quality makes it challenging to identify many proteins. Although protein identification using MS/MS yields higher-quality data, interpreting these data with this method requires a significant amount of time [6].

## 6. Conclusion

Proteomics is a growing technology that plays a crucial role in the area of biomedical sciences. Currently, it is applied for drug and vaccine discovery, stem cell study, personalized medicine, and research and diagnosis of infectious and noninfectious diseases. Nowadays, the most commonly and widely used proteomics techniques include MS and protein microarray. In proteomics, analysis of low-abundance proteins remains a major challenge and it is still not feasible to study proteins on a scale equivalent to that of the nucleic acids. Moreover, the current techniques have limitations in their capacity; some of them are not high-throughput, some are time consuming during data acquisition or analysis and data interpretation, and others scarify the quality of data and many proteins cannot be identified. Therefore, new methods must be devised for low-abundance protein isolation. New computer algorithms are needed to allow more accurate analysis and interpretation of proteomics data.

## Data Availability

The data supporting this review are from previously reported studies, which have been cited.
